# Lessening Organ dysfunction with VITamin C (LOVIT): protocol for a randomized controlled trial

**DOI:** 10.1186/s13063-019-3834-1

**Published:** 2020-01-08

**Authors:** Marie-Hélène Masse, Julie Ménard, Sheila Sprague, Marie-Claude Battista, Deborah J. Cook, Gordon H. Guyatt, Daren K. Heyland, Salmaan Kanji, Ruxandra Pinto, Andrew G. Day, Dian Cohen, Djillali Annane, Shay McGuinness, Rachael Parke, Anitra Carr, Yaseen Arabi, Bharath Kumar Tirupakuzhi Vijayaraghavan, Frédérick D’Aragon, Élaine Carbonneau, David Maslove, Miranda Hunt, Bram Rochwerg, Tina Millen, Michaël Chassé, Martine Lebrasseur, Patrick Archambault, Estel Deblois, Christine Drouin, François Lellouche, Patricia Lizotte, Irene Watpool, Rebecca Porteous, France Clarke, Nicole Marinoff, Émilie Belley-Côté, Brigitte Bolduc, Scott Walker, John Iazzetta, Neill K. J. Adhikari, François Lamontagne, on behalf of the Canadian Critical Care Trials Group

**Affiliations:** 1 Centre de Recherche du Centre Hospitalier Universitaire de Sherbrooke, Sherbrooke, QC Canada; 2 Division of Orthopaedic Surgery, Department of Surgery, McMaster University, Hamilton, ON Canada; 3 Department of Health Research Methods, Evidence, and Impact, McMaster University, Hamilton, ON Canada; 4 Centre de Recherche du Centre Hospitalier Universitaire de Sherbrooke Sherbrooke and Université de Sherbrooke, Sherbrooke, QC Canada; 5 Department of Critical Care Medicine, Queen’s University, Kingston, ON Canada; 6 Departments of Pharmacy and Critical Care, The Ottawa Hospital and Clinical Epidemiology Program, The Ottawa Hospital Research Institute, Ottawa, ON Canada; 7 Department of Critical Care Medicine, Sunnybrook Health Sciences Centre, Toronto, ON Canada; 8 Kingston General Health Research Institute, Kingston, ON Canada; 9 No institutional affiliation, No institutional affiliation, Sherbrooke, QC, Canada; 10 General Intensive Care Unit, Raymond Poincaré Hospital (AP-HP), Lab Inflammation & Infection, U1173 University Paris Saclay-UVSQ/INSERM, Garches, France; 11 Cardiothoracic and Vascular ICU, Auckland City Hospital, Auckland, New Zealand; 12 Medical Research Institute of New Zealand, Wellington, New Zealand; 13 School of Nursing, The University of Auckland, Auckland, New Zealand; 14 Department of Pathology and Biomedical Science, University of Otago, Christchurch, New Zealand; 15 College of Medicine, King Saud Bin Abdulaziz University for Health Sciences, King Abdullah International Medical Research Center, Riyadh, Saudi Arabia; 16 Department of Critical Care Medicine, Apollo Hospitals, Chennai, India; 17 Department of Critical Care Medicine, Queen’s University and Kingston Health Sciences Centre, Kingston, ON Canada; 18 Department of Medicine and Department of Health Research Methods, Evidence, and Impact, McMaster University, Hamilton, ON Canada; 19 Juravinski Hospital, Hamilton, ON Canada; 20 Centre de Recherche du Centre Hospitalier Universitaire de Montréal, Montréal, QC Canada; 21 Faculté de Médecine, Université Laval, Québec City, QC Canada; 22 Département des soins intensifs du Centre intégré de santé et des services sociaux de Chaudière-Appalaches (Secteur Alphonse-Desjardins), Lévis, QC Canada; 23 Department of Medecine, Université Laval and Centre de recherche de l’Institut universitaire de cardiologie et de pneumologie de Québec, Québec City, QC Canada; 24 Institut Universitaire de Cardiologie et de Pneumologie de Québec, Québec City, QC Canada; 25 Ottawa Hospital Research Institute, Ottawa, ON Canada; 26 Department of Critical Care Research, St. Joseph’s Healthcare, Hamilton, ON Canada; 27 Division of Cardiology, Department of Medicine, McMaster University, Population Health Research Institute, Hamilton, ON Canada; 28 Centre Hospitalier Universitaire de Sherbrooke, Sherbrooke, QC Canada; 29 Department of Pharmacy, Sunnybrook Health Sciences Centre, Toronto, Canada and Leslie Dan Faculty of Pharmacy, University of Toronto, Toronto, ON Canada; 30 Department of Pharmacy, Sunnybrook Health Sciences Centre, Toronto, ON Canada; 31 Interdepartmental Division of Critical Care Medicine and Institute for Health Policy, Management, and Evaluation, University of Toronto, Toronto, ON Canada

**Keywords:** vitamin C, sepsis, septic shock, biomarkers, randomized controlled trial

## Abstract

**Background:**

Sepsis is a health problem of global importance; treatments focus on controlling infection and supporting failing organs. Recent clinical research suggests that intravenous vitamin C may decrease mortality in sepsis. We have designed a randomized controlled trial (RCT) to ascertain the effect of vitamin C on the composite endpoint of death or persistent organ dysfunction at 28 days in patients with sepsis.

**Methods:**

LOVIT (Lessening Organ dysfunction with VITamin C) is a multicenter, parallel-group, blinded (participants, clinicians, study personnel, Steering Committee members, data analysts), superiority RCT (minimum *n* = 800). Eligible patients have sepsis as the diagnosis for admission to the intensive care unit (ICU) and are receiving vasopressors. Those admitted to the ICU for more than 24 h are excluded. Eligible patients are randomized to high-dose intravenous vitamin C (50 mg/kg every 6 h for 96 h) or placebo. The primary outcome is a composite of death or persistent organ dysfunction (need for vasopressors, invasive mechanical ventilation, or new and persisting renal replacement therapy) at day 28. Secondary outcomes include persistent organ dysfunction-free days to day 28, mortality and health-related quality of life at 6 months, biomarkers of dysoxia, inflammation, infection, endothelial function, and adverse effects (hemolysis, acute kidney injury, and hypoglycemia). Six subgroup analyses are planned.

**Discussion:**

This RCT will provide evidence of the effect of high-dose intravenous vitamin C on patient-important outcomes in patients with sepsis.

**Trial registration:**

clinicaltrials.gov, NCT03680274, first posted 21 September 2018.

## Background

The burden of sepsis is increasing worldwide. Defined as a dysregulated host immune response to infection that leads to organ failure and death [1], sepsis is the cause of up to 5.3 million deaths each year globally [2]. Currently, treatment options are limited to antimicrobials and organ-supportive care such as intravenous fluids, vasopressors, mechanical ventilation, and renal replacement therapy (RRT). In the absence of effective therapies targeting the dysregulated immune response, prolonged use of life-sustaining therapies is associated with substantial morbidity and mortality [3, 4]. Furthermore, in resource-constrained settings, life-sustaining therapies may be unavailable, and the prognosis of septic patients is grim [5]. This global burden led the World Health Assembly to adopt a resolution urging Member States and the World Health Organization Director-General to take action to reduce the burden of sepsis through improved prevention, diagnosis, and management [6].

Inflammation and oxidative stress are among the main mechanisms underlying sepsis-induced organ injury and death [7]. During sepsis, leukocytes produce reactive oxygen species to facilitate killing of pathogens. Normally, endogenous antioxidants contain this response and protect cells from oxidative damage. Vitamin C, a key circulating antioxidant [8], cannot be synthesized by humans and therefore requires exogenous intake. Moreover, many critically ill patients are vitamin C deficient and, even when they are not, sepsis further exhausts vitamin C stores [9, 10]. Low serum levels of ascorbic acid are associated with sepsis-induced organ failure and death [11]. Numerous preclinical studies have shown that, in addition to direct scavenging of oxygen radicals, vitamin C limits their production and restores endothelial function [12–14]. Vitamin C is also a cofactor in the synthesis of norepinephrine and vasopressin, hormones that are crucial to maintain adequate vascular tone for organ perfusion [15].

A recent pre-post single-centre study (*n* = 94) treated septic shock patients with a combination of intravenous (IV) vitamin C, hydrocortisone, and thiamine and reported a dramatic reduction in vasopressor requirements, organ failure, and hospital mortality (mortality 8.5% [combination therapy] vs. 40.4% [control]; adjusted odds ratio [OR] 0.13, 95% confidence interval [CI] 0.04–0.48) [16]. In 2014, Fowler et al. compared high-dose (50 mg/kg IV every 6 h for 4 days) and low-dose vitamin C (12.5 mg/kg IV every 6 h for 4 days) to placebo in a randomized controlled trial (RCT) of 24 patients with sepsis [17]. High-dose vitamin C was associated with a significant improvement in the slope of Sequential Organ Failure Assessment (SOFA) scores [18] over 96 h, implying faster resolution of organ dysfunction. In 2016, Zabet et al. conducted a 28-patient RCT of vitamin C (25 mg/kg IV every 6 h for 72 h) vs. placebo [19] and demonstrated that vitamin C reduced the mean (± SD) dose of norepinephrine during the study period (7.44 ± 3.65 μg/min vs. 13.79 ± 6.48 μg/min, *P* = 0.004). A meta-analysis [20] of both trials suggested that intravenous vitamin C monotherapy may reduce mortality (OR 0.21, 95%CI 0.04–1.05; *P* = 0.06), although conclusions were limited by imprecision and risk of bias (unclear allocation concealment) of one of the included trials [19]. More recently, a RCT of high-dose vitamin C vs. placebo in 167 patients with sepsis and acute respiratory distress syndrome showed no difference in the primary outcomes of change in organ failure or plasma biomarkers of inflammation and vascular injury [21]. This trial also found an improvement in the secondary outcome of 28-day mortality (risk difference, − 15%, 95%CI − 2% to − 31%, *P* = 0.03), but the result had a very low fragility index of 1 and was one of 46 secondary outcomes [22].

### Primary aim and hypothesis

The primary aim of the LOVIT (Lessening Organ dysfunction with VITamin C) RCT is to determine whether intravenous vitamin C, administered as an isolated intervention (not part of a combination of therapies) to patients with sepsis receiving a vasopressor, reduces a composite of mortality and persistent organ dysfunction [23] (defined as dependency on vasopressors, mechanical ventilation, or incident RRT) at 28 days after randomization, when compared to placebo.

## Methods

LOVIT is a multicentre, parallel-group, allocation-concealed, blinded (participants, clinicians, study personnel, members of the Executive and Steering Committees, and data analysts), superiority RCT. Table 1 shows a timeline of trial activities. The SPIRIT checklist is available in Additional file 2.

**Table 1:** LOVIT trial timeline

TIME POINTS	Study Period												
	Days	Days											Months
	Enrolment/Allocation	Post-Allocation											
	1	2	3	4	5–6	7	8–9	10	11–13	14	15–27	28	6 months
ENROLMENT:													
Eligibility screen	x												
Informed consent	x												
Allocation	x												
INTERVENTION:													
Vitamin C or placebo	x	x	x	x									
ASSESSMENTS:													
Baseline variables													
Source of infection	x												
Severity of illness (APACHE II score)	x												
Pre-existing comorbidities (Charlson comorbidity index, Clinical Frailty Score)	x												
Vitamin C level^a^	x												
Primary outcome													
Death or persistent organ dysfunction^b^												x	
Secondary outcomes													
Persistent organ dysfunction-free days in ICU, up to day 28^b^	x	x	x	x	x	x	x	x	x	x	x	x	
Mortality at 6 months													x
HRQoL (EQ-5D-5 L)													x
Global tissue dysoxia (serum lactate concentration)	x		x			x							
Organ function including renal function (SOFA score)	x	x	x	x		x		x		x		x	
Inflammation biomarkers (IL-1ß, TNF-α, CRP)	x		x			x							
Infection biomarkers (PCT)	x		x			x							
Endothelial injury biomarkers (TM, ANG-2)	x		x			x							
Occurrence of stage 3 AKI^c^	x	x	x	x	x	x	x	x	x	x	x	x	
Acute hemolysis	x	x	x	x	x	x	x	x	x	x	x	x	
Hypoglycemia^d^	x	x	x	x	x	x	x	x	x				
Other variables													
Protocol adherence^e^	x	x	x	x									
Co-interventions^f^	x	x	x	x	x	x	x	x	x	x		x	

*AKI* acute kidney injury, *ANG-2* angiopoietin-2, *APACHE II score* Acute Physiology and Chronic Health Evaluation II score, *CRP* C-reactive protein, *EQ-5D-5 L questionnaire* EuroQol-5D (EQ-5D), *HRQoL*: health-related quality of life, *ICU* intensive care unit, *IL-1ß* interleukin-1 beta, *PCT* procalcitonin, *SOFA score* Sequential Organ Failure Assessment score, *TM* thrombomodulin, *TNF-α* tumor necrosis factor-alpha

^a^Must be collected before the first dose of investigational product

^b^Dependency on mechanical ventilation, RRT, or vasopressors

^c^As defined by KDIGO (Kidney Disease: Improving Global Outcomes) criteria^22^

^d^Assessed by core lab (during the time participants receive the 16 doses of the investigational product and the 7 days following the last dose)

^e^Receipt of every planned dose according to schedule until completion of 96-h treatment protocol, samples collected per protocol instructions, vitamin C baseline collected before the first dose of investigational product

^f^Administration of mechanical ventilation, RRT, vasopressors, corticosteroids, thiamine, intravenous fluids, blood products, sedatives, and antimicrobials. Daily data until ICU discharge or 28 days (whichever comes first)

### Study setting

This trial is centrally coordinated by the Unité de Recherche Clinique et Épidémiologique (URCE) at the Centre de Recherche du Centre Hospitalier Universitaire de Sherbrooke, Sherbrooke, QC, Canada. The URCE also oversees the activities of the LOVIT core laboratory (i.e. storage and analysis of blood and urine samples). The CLARITY Research Group, Department of Health Research Methods, Evidence, and Impact, McMaster University, Hamilton, ON, Canada constructed and will maintain the randomization system and the iDataFax (DF/Net Research, Inc., Seattle, WA, USA) electronic data capture (EDC) system. The LOVIT trial will be conducted in up to 25 adult intensive care units (ICUs) in Canada and other countries.

### Inclusion criteria

Patients will be included if they are:

1. ≥18 years old;
2. admitted to an ICU with proven or suspected infection as the main diagnosis;
3. treated with a continuous intravenous vasopressor infusion (norepinephrine, epinephrine, vasopressin, dopamine, or phenylephrine) at the time of eligibility assessment and at randomization.

### Exclusion criteria

Patients are excluded for the following reasons:

1. > 24 h since ICU admission;
2. known glucose-6-phosphate dehydrogenase (G6PD) deficiency;
3. pregnancy;
4. known allergy to vitamin C;
5. known kidney stones within the past 1 year;
6. received any intravenous vitamin C during current hospitalization, unless incorporated as part of parenteral nutrition;
7. expected death or withdrawal of life-sustaining treatments within 48 h;
8. previously enrolled in this study;
9. enrolled in a trial for which co-enrolment is not possible (to be determined on a case-by-case basis by discussion with the other trial’s principal investigators).

### Rationale for eligibility criteria

Eligibility criteria are broad and consider most patients admitted to the ICU with a primary diagnosis of sepsis and receiving vasopressor therapy. We decided not to mandate the Sepsis-3 definition of septic shock [1], which also includes an elevated lactate level, to facilitate enrollment in and enhance generalisability to settings where lactate measurements are not routinely available. By including patients admitted to the ICU for ≤24 h, we exclude patients admitted for another reason who develop sepsis secondarily and whose prognosis may thus be more related to the primary reason for ICU admission. By limiting eligibility to patients who are dependent on vasopressors, we are including those with the highest baseline risk of the primary outcome and therefore those with the most to gain from an intervention targeted to increase synthesis of or sensitivity to endogenous catecholamines [15]. The exclusion of patients with known G6PD deficiency is related to the risks of hemolysis [24]; reported cases have occurred with higher doses than used in LOVIT [25]. Patients with a previous history of kidney stones may be at increased risk of stone formation with vitamin C due to oxaluria [26].

### Recruitment strategy and approach for consent

Local research personnel will screen for potentially eligible patients in ICUs and approach eligible patients directly if they are able to consent. If an eligible patient is not capable, research personnel will approach the surrogate decision-maker (SDM) to obtain consent in person, or by telephone if the SDM is unavailable. Alternatively, the patient will be randomized and consent will be obtained subsequently under a deferred consent model, where permitted by the site Research Ethics Board (REB) and as per individual country requirements concerning informed consent in medical research. The first dose of the study drug must be administered within 4 h of randomization and the blood samples drawn before giving the first dose of study drug.

### Ethics

The Coordinating Centre and all participating clinical sites will receive local REB approval prior to commencing participant enrolment. Before initiating the trial, each clinical site will provide the Coordinating Centre with a copy of their local REB approval letter and approved informed consent form (sample in Additional file 3). Consent is requested for future laboratory analyses that may arise from this protocol. Any required protocol amendments will be submitted to each REB and disseminated to all investigators after approval from Health Canada, the regulatory agency for drug studies in Canada.

There is no compensation for harm suffered from trial participation; details on data collection for adverse events (AEs) are given below. Patients enrolled in this trial are critically ill and all care will be provided by intensive care clinicians. There is no provision for post-trial care other than usual clinical care for ICU patients.

### Randomization

A web-based randomization service (www.randomize.net) will be available 24 h per day, 7 days per week. Trial participants will be randomized in a 1:1 ratio to vitamin C or matching placebo using permuted blocks of undisclosed and variable size stratified by clinical site.

### Trial interventions

The experimental intervention is intravenous vitamin C administered in bolus doses of 50 mg/kg actual body weight mixed in a 50-mL solution of either dextrose 5% in water (D5W) or normal saline (0.9% NaCl), given every 6 h for 96 h (i.e. 200 mg/kg/day and 16 doses in total), as long as the patient remains in the ICU. For patients weighing ≥125 kg, 12 ml of D5W or 0.9% NaCl is added to the bag to keep the concentration of vitamin C in the range tested in a stability study [27]. For patients weighing ≥150 kg, the weight is considered as 150 kg to calculate the dose. Each dose is administered over 30–60 min, except for participants > 120 kg, for whom the infusion time is prolonged so that the rate does not exceed 100 mg/min. Once mixed in either solution, covered bags may be stored at 4 °C for a maximum of 9 days. This procedure was validated by a stability study, which found that at concentrations between 37 and 92 mg/mL, vitamin C is physically and chemically stable for at least 14 days at 4 °C, when protected from light [27]. Notwithstanding the results of the stability study, the 9-day maximum is derived from regulations concerning intravenous drug administration in Canada [28]. Because the infusion time is short, covering medication bags and tubing is not required during the infusion.

Participants in the control arm will receive D5W or 0.9% NaCl in a volume to match the vitamin C. Placebo will be infused over the same period as per the instructions for vitamin C and is identical in colour and other physical properties to vitamin C.

At each clinical site, the preparation of solutions administered to participants will be the responsibility of the unblinded study pharmacist, who is not involved in patient care. Both vitamin C and placebo (D5W or 0.9% NaCl) will be purchased by sites from their usual distributor. Alternative arrangements, such as shipping of vitamin C from Canada, may be considered for any international sites.

### Rationale for interventions

We selected a high-dose vitamin C regimen since this may be associated with the highest likelihood of demonstrating an effect, if one exists [17]. We considered, but rejected, the idea of evaluating the effect of a combined experimental intervention consisting of vitamin C, corticosteroids, and thiamine. We believe that existing data do not mandate co-administration of vitamin C with other interventions and that a triple-dummy design would be overly complicated and may compromise equipoise given recent guidelines in favor of corticosteroid therapy for sepsis [29]. Although this trial will evaluate vitamin C monotherapy, we will collect information on clinicians’ use of co-interventions including thiamine and corticosteroids.

### Co-interventions

All co-interventions are at the discretion of the treating physicians, who will be blinded to treatment assignment. However, we will record use of relevant co-interventions – ventilation, RRT, vasopressors, corticosteroids, thiamine, nutrition, blood products, analgesia and sedation, and antimicrobials – to detect and report any imbalances between treatment groups.

Unblinded administration of IV open-label vitamin C, except as part of parenteral nutrition, is discouraged and will constitute a protocol violation.

### Blinding

Blinding will be achieved by using indistinguishable vitamin C and placebo products.

Pharmacists and technicians preparing the study drug (vitamin C or placebo) at each participating site will be unblinded. If a clinical situation calls for unblinding of other personnel, as assessed on a case-by-case basis, a designated unblinded Coordinating Centre research coordinator will apply the unblinding procedure (Table 2). Unblinded pharmacists will not be authorized to independently unblind additional personnel.

**Table 2:** Unblinding of clinical site personnel for emergency medical management

1. In the event of a medical emergency that directly affects the health status of the participant, it may become necessary to unblind allocation status to determine the specific treatment the participant has received while enrolled in the study. A medical emergency is defined as an event which necessitates immediate attention regarding the treatment of a participant.	
2. Clinical site personnel (e.g. local principal investigators, co-investigators, and research coordinators) will contact one of the LOVIT principal investigators if they believe that unblinding of a study participant is medically necessary.	
3. The LOVIT principal investigator will discuss the participant’s medical event with the clinical site personnel and determine if it is necessary to unblind her/him to the participant’s treatment allocation. At no time will the participant’s health be compromised or medical treatment delayed.	
4. Once approved, the principal investigator or coordinating centre project leader will contact the designated unblinded research coordinator at the coordinating centre, who will provide the clinical site personnel with the participant’s vitamin C or placebo allocation. This information should be provided by telephone to reduce the risk of unblinding additional team members.	
5. The unblinded research coordinator will complete the LOVIT unblinding log. The unblinded research coordinator may contact the research coordinator at the clinical site to request any additional information required to complete this log.	
6. The unblinded research coordinator is not to unblind the principal investigators or any blinded members of the LOVIT team unless deemed necessary by the principal investigator. Similarly, clinical site personnel are also not to unblind any other members of the LOVIT study team (including the Methods Centre research coordinator, principal investigators, or any clinical site research personnel who were not unblinded) unless deemed necessary by the principal investigator(s).	
7. LOVIT personnel must keep all information related to the individual unblinding cases confidential.	
8. All cases of unblinding must be documented, including clinical site ID, study ID, date of unblinding, parties unblinded, and reason for unblinding.	

### Primary outcome measure

The primary outcome will be a composite of death or persistent organ dysfunction (defined as dependency on vasopressors, mechanical ventilation, or new and persisting RRT) at day 28 after randomization [23]. Patients with pre-existing end-stage renal disease receiving RRT before the index hospitalization will not meet criteria for persistent organ dysfunction on the basis of ongoing RRT.

### Secondary outcome measures

Note that day 1 refers to the day of randomization.

Efficacy outcomes will be:

1. Persistent organ dysfunction-free days in ICU, defined as number of days alive and not dependent on vasopressors, mechanical ventilation, or RRT, up to day 28;
2. Mortality at 6 months;
3. Health-related quality of life (HRQoL) in 6-month survivors as assessed by the EuroQol-5D-5 L (EQ-5D-5 L) [30], a standardized questionnaire of health status developed to provide a simple, generic measure of health for clinical and economic appraisal. Health status is measured in terms of mobility, self-care, usual activities, pain/discomfort, and anxiety/depression. Respondents also evaluate their overall health status using a 100-point scale;
4. Global tissue dysoxia assessed at days 3 and 7 of hospitalization by serum lactate concentration [31]. This will be assessed using liquid chromatography coupled with tandem mass spectrometry (LC-MS/MS);
5. Organ function (including renal function) assessed by the SOFA score [18] at days 2, 3, 4, 7, 10, 14, and 28;
6. Inflammation at days 3 and 7 of hospitalization, assessed by serum interleukin-1 beta, tumor necrosis factor-alpha, and C-reactive protein levels, measured by Luminex (Luminex Corp., Austin, TX, USA);
7. An infection biomarker at days 3 and 7 of hospitalization, measured by serum procalcitonin [17] using an enzyme-linked immunosorbent assay;
8. Endothelial injury assessed at days 3 and 7 of hospitalization by serum thrombomodulin [17] and angiopoietin-2 [32], measured by Luminex.

Safety outcomes will be:

9) Stage 3 acute kidney injury (AKI) as defined by Kidney Disease-Improving Global Outcomes criteria [33] using either creatinine or urine output criteria, at any time during the ICU stay;
10) Acute hemolysis, ascertained until 12 h after the last dose of study medication, defined as clinician judgment of hemolysis, as recorded in the chart, or a hemoglobin drop of at least 25 g/L within 24 h of a dose of study drug and 2 of the following: reticulocyte count > 2 times upper limit of normal; haptoglobin less than lower limit of normal; indirect (unconjugated) bilirubin > 2 times upper limit of normal; or lactate dehydrogenase > 2 times upper limit of normal. Normal values are as defined at each participating centre’s laboratory. Severe hemolysis is defined as hemoglobin < 75 g/L, at least 2 of the above criteria and the requirement for transfusion of at least 2 units of packed red blood cells.
11) Hypoglycemia, defined as a blood glucose level measured in the hospital core laboratory of less than 3.8 mmol/L. Vitamin C therapy may be associated with false hyperglycemic readings when point-of-care glucometers are used to measure blood glucose [34]. Accordingly, blood glucose can only be measured by one of the following three methods: hospital core laboratory instruments; a point-of-care arterial blood gas machine whose glucose measurement has been validated in the setting of high blood concentration of ascorbic acid; and point-of-care StatStrip glucometers (Nova Biomedical, Waltham, MA, USA), whose measurements have been shown to be accurate in the presence of high blood concentration of ascorbic acid. Blood glucose measurements in patients receiving insulin or oral hypoglycemic agents will be monitored as described above up to 7 days after the last dose of the investigational product. Because hyperglycemic readings may prompt iatrogenic hypoglycemic episodes if insulin or oral hypoglycemic agents are administered, hypoglycemic events will be reported as a safety outcome.

### Adverse events

In the context of critical illness, all patients eligible for the LOVIT trial are at risk of AEs. Following Canadian guidelines for AE reporting in academic critical care trials [35], expected AEs (death, stage 3 AKI, hemolysis, hypoglycemia), whether severe or not, are pre-specified trial outcomes and will not be reported separately as AEs.

In contrast, unexpected AEs that are serious (i.e. fatal, life-threatening, prolonging hospital stay, resulting in persistent or significant disability or incapacity, or constituting an important medical event according to the local principal investigator) and considered by the local principal investigator to be at least possibly related to trial procedures will be reported to the Coordinating Centre within 24 h of becoming aware of the event. The Coordinating Centre will inform applicable regulatory agencies of serious AEs possibly related to the trial procedures within 15 days of receiving the report if the AE was neither fatal nor life-threatening, or within 7 days if it was fatal or life-threatening.

Within 8 days of informing the regulatory agency of a serious unexpected AE, the Coordinating Centre will submit to the regulatory agency a report including an assessment of the importance and implication of findings. This information will be updated with the patient’s final status.

### Additional analyses

Conditional upon obtaining the participants’ consent for future analyses, blood and urine specimens collected in the context of this trial will be stored for future analyses. These analyses will not include genetic testing.

### Data collection

Clinical (non-biomarker) data will be obtained from participants’ medical records. Local research teams will be responsible for data collection while participants are still hospitalized. To ensure uniform use of the follow-up questionnaire and reduce burden on local research teams, telephone follow-up at 6 months will be conducted by the Coordinating Centre research team, or by a designated centre in each country for international sites.

We will collect the following data:

1. Baseline (day 1): patient demographics, biomarkers (as listed in secondary outcomes), source of infection, severity of illness (APACHE II [Acute Physiology and Chronic Health Evaluation II] score [36]), organ function, including renal function (SOFA score [18]), pre-existing comorbidities as defined in the Charlson comorbidity index [37], and Clinical Frailty Scale estimated within 3 months of the current admission [38–40]. Baseline blood samples will be collected before the first dose of study product. We will use baseline ascorbic acid level for subgroup analysis, described below.
2. Daily data until ICU discharge or 28 days (whichever comes first): protocol adherence (administration of every planned dose until completion of the treatment protocol or ICU discharge, whichever comes first), co-interventions (ventilation, RRT, vasopressors, corticosteroids, thiamine, nutrition, blood products, analgesia and sedation, and antimicrobials).
3. Days 3 and 7 (if in ICU): blood samples for biomarkers (as listed in secondary outcomes); urine samples (for oxalate measurements if requested by the Data and Safety Monitoring Committee [DSMC]).
4. Days 2, 3, 4, 7, 10, 14, and 28 (if in ICU): SOFA score [18].
5. 28 days: death or persistent organ dysfunction (defined as dependency on mechanical ventilation, new and persistent RRT, or vasopressors) [23].
6. 6 months: mortality and HRQoL [30]. We will determine the date of death for patients that have died by 6 months.

To minimize participant discomfort and bedside teams’ workload, study sample collection will coincide as much as possible with drawing of blood for clinical reasons.

The LOVIT core laboratory will measure the biomarkers listed as secondary outcomes. Individual sites will ship all blood samples to the LOVIT core laboratory for analysis. Treating teams will remain blinded to the results of the biomarker assays performed as part of the study but will have access to all blood tests obtained for clinical reasons. Local laboratory results will be used as required for the components of the SOFA score. In case of missing data, SOFA scores will be imputed as pre-specified in the statistical analysis plan before unblinding the trial.

### Study follow-up and cohort retention

Once a patient is enrolled in the trial, the clinical site will make every reasonable effort to follow the participant for the entire duration of the study period. To minimize loss to follow-up at 6 months, consent forms will include permission to collect alternate contact information. If necessary, the Coordinating Centre will request the assistance of the local research team.

Participants may discontinue participation in the LOVIT trial at any time. If a participant wishes to withdraw consent, we will use the following strategies to minimize the impact on the trial, while respecting autonomy. We will seek a better understanding of the participant’s wishes and offer the following alternatives to complete withdrawal, which would include no further study drug exposure, data deletion, and sample destruction:

1. Discontinue study drug but allow data collection (clinical data, sample collection, telephone follow-up);
2. Discontinue study drug, in-person follow-up, and sample collection but allow telephone follow-up;
3. Discontinue study drug, sample collection, and in-person and telephone follow-up, but allow access to medical records.

### Intention to treat and ineligible participants

We will adhere to the intention-to-treat principle, and data from participants will be analyzed in the group to which they will have been allocated irrespective of protocol adherence. Reasons for protocol deviations, should they arise, will be recorded. If ineligible participants are randomized, we will allow post-randomization exclusions if 1) the information about ineligibility was available at randomization; 2) two members of the Steering Committee blinded to allocation agree that the participant was mistakenly randomized after review of information from medical records available at the time of randomization; 3) participants did not receive the assigned intervention; and 4) participants remain blinded to their allocation [41, 42]. Verification from the Coordinating Center is needed for official confirmation of participant withdrawal.

### Reducing bias

Risk of bias will be reduced by concealed randomization using variable and undisclosed blocks. Due to blinding of all relevant parties, decisions to discontinue life-sustaining therapies and other outcomes that require subjective assessments (e.g. HRQoL) will not be affected by individually held beliefs regarding the effects of vitamin C. In addition, to ensure consistent measurement of biomarkers in this trial, samples will be collected, frozen on-site and then shipped to the LOVIT core laboratory in Sherbrooke, QC, Canada, where they will be stored, batched and processed at the end of the trial. Finally, we will record co-interventions to detect performance bias.

### Statistical analyses

#### Sample size

We determined a minimum sample size of 800 participants based on the following assumptions. We established that an absolute difference of 10% in the composite outcome of death or persistent organ dysfunction (15 to 25% relative risk reduction) is plausible [16, 19] and sufficiently large to change practice. Based on recent clinical trials in a similar population [43], the risk of 28-day persistent organ dysfunction or mortality in the control arm is expected to be approximately 50%. By enrolling 385 evaluable patients per arm, the study will have 80% power to detect a 10% absolute risk reduction (from 50% to 40%, which corresponds to a 20% relative risk reduction). To account for consent withdrawal and loss to follow-up, we will enroll 400 patients per arm. If the control event rate were greater or lower than 50% and the absolute treatment effect remained 10%, statistical power would increase.

If international sites join the trial, the sample size and power to detect a difference in the primary outcome may increase (see section on International collaborations and sample size flexibility).

#### Data analysis

All analyses will be described in detail in a separately published statistical analysis plan. Statistical tests will be two-sided, and all analyses will be performed using SAS 9.4 (SAS Institute, Cary, NC, USA) or later. *P* < 0.05 will be interpreted as statistically significant.

For the primary analysis, we will compare the proportion of patients meeting the composite endpoint of persistent organ dysfunction or death at day 28 between groups using a model that accounts for the stratification variable [44]. The testing strategy for the primary outcome will maintain an overall two-sided type I error of 0.05. We will conduct sensitivity analyses adjusting for important pre-specified baseline clinical variables associated with the primary outcome [45]. Subgroup analyses will use models with terms for subgroup indicators and their interaction with treatment.

#### Pre-specified subgroup analyses

The LOVIT trial will evaluate the effect of vitamin C in the six subgroups defined at baseline by age (< 65 vs. ≥65 years), sex, frailty (Clinical Frailty Scale 1–4 vs. ≥5), severity of illness (quartiles of predicted risk of death from baseline APACHE II score), strict Sepsis-3 definition of septic shock (vasopressor therapy required to maintain a mean arterial pressure of 65 mmHg and lactate ≥2 mmol/L, vs. vasopressor need alone), and baseline ascorbic acid level (assuming sufficient variation). We hypothesize that vitamin C is more beneficial in elderly patients, in those with greater frailty and illness severity at baseline, those who meet strict criteria for septic shock, and in those with lower baseline ascorbic acid levels.

#### Interim analysis plan

The DSMC will review data on all possibly related serious AEs, hemolysis, stage 3 AKI, and hypoglycemia after the enrollment, 28-day follow-up, and data cleaning of 250 and 530 patients. If the one-sided *P* value is < 0.1 (in the direction of harm in the vitamin C arm) for any of the three safety outcomes, an interim two-sided analysis of the primary outcome will be conducted. The DSMC may also request an analysis of the primary outcome at any time. This analysis will generate a conditional power for showing statistically significant efficacy in the final analysis of the primary outcome, assuming that the group-specific event rates observed to date remain the same in the total sample size. If the conditional power for efficacy is < 20%, in the context of a one-sided *P* < 0.1 for any of the safety outcomes, then the DSMC may recommend stopping the trial to the Steering Committee. The DSMC may make a similar recommendation even if these exact thresholds are not met, based on its interpretation of the balance between safety and efficacy.

Following the recent RCT of high-dose vitamin C [21], the DSMC has requested an interim analysis of 28-day mortality after the enrollment of 530 patients, and may recommend stopping the trial to the Steering Committee if two-sided *P* < 0.001.

### Registration

An initial ‘no objection’ letter was received from Health Canada on 5 September 2018 (HC6–24-c219212), and the trial was registered on www.clinicaltrials.gov on 21 September 2018 (NCT03680274).

### Data management

The paper or electronic case report forms (CRFs) are the primary data collection tool for the study. All data requested on the CRF must be recorded either on paper CRFs or on the electronic CRFs within the secure iDataFax EDC system. If the data are first collected on paper CRFs, site research personnel will subsequently transfer all data into iDataFax by direct entry. CLARITY research staff are responsible for programming and maintaining the database. With support from CLARITY, Coordinating Centre staff are responsible for trial monitoring.

### Monitoring

Quality control measures include 1) on-site training of research and clinical personnel; 2) standard operating procedures to guide storage, preparation and administration of the study drug as well as processing, storage, and shipping of blood and urine samples; 3) ongoing assessment of trial management metrics (monthly screening logs, weekly reports [site enrolment rate, protocol adherence regarding study drug administration and study samples, length of ICU stay, vasopressor treatment duration, in-ICU and in-hospital mortality, mortality and persistent organ dysfunction at day 28]) and periodic feedback to the clinical sites on performance (recruitment, protocol adherence), with benchmarking from other sites; 4) site monitoring visits (remotely or in person for two of the first five participants and 10% of the subsequent participants); 5) ongoing review of missing data and outliers; and 6) rapid dissemination of responses to frequently asked questions via our study website (http://lovit.ccctg.ca/) and monthly newsletter. Coordinating Centre staff and the Principal Investigators are available at all times to answer study-related questions.

### Trial oversight

#### Executive Committee

The Executive Committee is comprised of Neill KJ Adhikari and François Lamontagne (co-principal investigators), Marie-Claude Battista (core laboratory), Marie-Hélène Masse, Julie Ménard, and Sheila Sprague (co-project leaders). The Executive Committee is responsible for day-to-day management and is accountable to the Steering Committee.

#### Steering Committee

The Steering Committee consists of Executive Committee members and additional clinical, methodological, and statistical experts, in addition to a patient representative (listed in Additional file 1). It meets quarterly by teleconference. The Steering Committee provides guidance and direction to the trial.

#### Data Safety Monitoring Committee

The DSMC is independent of the study investigators and responsible for safeguarding the interests of study participants, assessing the safety and efficacy of study procedures, and monitoring the overall conduct of the study. The DSMC members have extensive trial experience and include a senior methodologist who has served as Chair on numerous DSMCs for international RCTs, a senior biostatistician, and a clinician-scientist in intensive care (Additional file 1). The DSMC will periodically review enrolment and demographic summaries, protocol deviations, and serious AEs. In accordance with a pre-specified DSMC Charter, the DSMC will advise the Executive and Steering Committees on any concerns related to participant safety and trial conduct. After each meeting, the DSMC will make a recommendation for the study to continue as designed, termination, continuation with major or minor modifications, or temporary suspension of enrolment until some uncertainty is resolved.

### International collaborations and sample size flexibility

LOVIT is designed as a living umbrella RCT, a concept that permits the inflation of the overall sample size as international collaborations augment trial capacity. This model increases the efficiency of clinical trial research by increasing the probability that a single trial will be sufficiently powered to detect a minimally clinically important treatment effect. Because the same core data are collected in every country, the need to harmonize heterogeneous datasets is avoided. However, each national lead is free to seek additional funding and to add secondary objectives to the main protocol.

Following this model, the current protocol, CRFs, Operation Manual, and other trial-related documents were shared with collaborators from other countries who have agreed to join the LOVIT team of investigators, thus creating the International LOVIT Collaborative. Our collaborators have been invited to become the LOVIT national leads in their respective countries and recruit patients in the current protocol (*n* = 800), secure additional resources to enroll more patients, or both. Our terms of engagement stipulate that for participants in the current protocol (*n* = 800), specimens for baseline measurements of ascorbic acid and lactate must be collected and shipped to the LOVIT core laboratory. If our international collaborators secure resources to recruit additional patients, the clinical data described in this protocol will be stored in the Canadian database so that the primary analysis may include all participants (*n* > 800). Additional clinical data pertaining to any embedded studies may be kept in the country of origin. These add-on studies will focus on outcomes or subgroups not described herein and will be reported separately, coauthored by the co-principal investigators of this parent protocol.

### Dissemination

Results of the LOVIT trial will be presented at international conferences, published in peer-reviewed journals, and disseminated via social media platforms and discussion fora managed by partner organizations.

The LOVIT protocol is freely accessible via this publication. The principal investigators, project leaders, and study statisticians will have access to the full trial dataset; there are no contractual limitations to such access. The LOVIT policy on authorship and sub-studies that would require access to the trial dataset is available in Additional file 4. Other requests for access to the participant-level dataset and statistical code will be considered by the Steering Committee after publication of primary results and planned secondary studies by co-investigators.

## Discussion

Vitamin C, an inexpensive and readily available intervention, may be a life-saving therapy in sepsis. If proven effective, vitamin C could be used worldwide to improve outcomes in high- and low-income settings alike.

The LOVIT protocol constitutes a rigorous assessment of the effect of vitamin C monotherapy on patient-important outcomes in critically ill patients with sepsis. We are working with site principal investigators to convince clinicians to enroll eligible patients in LOVIT rather than to prescribe this treatment to patients on the basis of current data, which constitutes low-certainty evidence. Once completed, the LOVIT International Collaborative is committed to harmonize data from LOVIT and other trials of intravenous vitamin C in an individual patient data meta-analysis.

### Trial status

The current protocol is version 5, last updated 19 September 2019. Participant recruitment began on 14 November 2018 and is scheduled to continue for 36 months, until approximately 13 November 2021. The database will be locked after the last enrolled patient completes the 6-month follow-up, and 6 additional months will be required to address remaining data queries and to finalize the analyses.

*Contact information for trial sponsor*

François Lamontagne (francois.lamontagne@usherbrooke.ca)

Université de Sherbrooke

3001 12^e^ Avenue Nord

Sherbrooke QC J1H 5 N4 Canada

## Supplementary information

**Additional file 1.** LOVIT contributors.

**Additional file 2.** SPIRIT checklist.

**Additional file 3.** Model informed consent form.

**Additional file 4.** Authorship and substudy guidelines.

## Data Availability

Data sharing is not applicable to this article as no datasets were generated or analysed during the current study.

## Abbreviations

0.9% NaCI: 0.9% Sodium chloride
AE: Adverse event
AKI: Acute kidney injury
APACHE II: Acute physiology and chronic health evaluation II
CI: Confidence interval
CRF: Case report form
D5W: Dextrose 5% in water
DSMC: Data and Safety Monitoring Committee
EDC: Electronic data capture
G6PD: Glucose-6-phosphate dehydrogenase
HRQoL: Health-related quality of life
ICU: Intensive care unit
IV: Intravenous
LOVIT: Lessening Organ dysfunction with VITamin C
OR: Odds ratio
RCT: Randomized controlled trial
REB: Research Ethics Board
RRT: Renal replacement therapy
SD: Standard deviation
SDM: Surrogate decision-maker
SOFA: Sequential (sepsis-related) organ failure assessment
URCE: Unité de recherche clinique et épidémiologique
