# Supplementary material for: Lessening Organ dysfunction with VITamin C (LOVIT): protocol for a randomized controlled trial
Source: Trials. 2020 Jan 8;21:42. doi: 10.1186/s13063-019-3834-1 (PMC6950903; doi:10.1186/s13063-019-3834-1)
Supplement: Supplementary file 1 — Additional file 1. LOVIT contributors. [file 13063_2019_3834_MOESM1_ESM.pdf]

Additional File 1 LOVIT team members, including research personnel at clinical sites active at time of submission of this manuscript

Executive Committee
Neill KJ Adhikari (PI, co-chair), François Lamontagne (PI, co-chair), Marie-Hélène Masse (PL), Julie Ménard (PL), Sheila Sprague (PL)

Steering Committee
Neill KJ Adhikari (co-chair), François Lamontagne (co-chair), Marie-Claude Battista, Dian Cohen, Deborah Cook, Andrew Day, Gordon Guyatt, Daren Heyland, Salmaan Kanji, Marie-Hélène Masse, Julie Ménard, Ruxandra Pinto, Sheila Sprague

Data and Safety Monitoring Committee
Andreas Laupacis (chair), Lauren Griffith, Scott Halpern

Coordinating Centre Personnel
Marie-Claude Battista, Francis Malenfant, Marie-Hélène Masse, Julie Ménard, Louise Robert-Petit, Amélie Têtu, Marie-Ève Thibault, Sheila Sprague

Participating Clinical Sites Personnel
CIUSSS de l'Estrie – Centre Hospitalier Universitaire de Sherbrooke
François Lamontagne (PI), Frédérick D'Aragon (Co-I), Marc-André Leclair (Co-I), Michaël Mayette (Co-I), Yannick Poulin (Co-I), Hector Quiroz-Martinez (Co-I), Charles St-Arnaud (Co-I), Élaine Carbonneau (RC), Line Côté (RN), Marilène Ladouceur (RN), Joannie Marchand (RA), Marie-Hélène Masse (RC), Noémie Turcotte (RA)

CISSS - Chaudière-Appalaches
Patrick Archambault (PI), Christine Drouin (Co-I), Estel Deblois (RC), Krystel Gonthier (RN), Lise Labrie (RN)

Centre Hospitalier de l'Université de Montréal
Michaël Chassé (PI), Pierre Aslanian (Co-I), François Martin Carrier (Co-I), Martin Girard (Co-I), Antoine Halwagi (Co-I), Martine Lebrasseur (RC), Fatna Benettaib (RC), Dounia Boumahni (RC), Marie-Ève Cantin (RA), Ali Ghamraoui (RC), Maya Salame (RC)

Institut Universitaire de Cardiologie et de Pneumologie de Québec
François Lellouche (PI), Ying Tung Sia (Co-I), Mathieu Simon (Co-I), Patricia Lizotte (RC), Pierre-Alexandre Bouchard (RC)

CIUSSS de l'Est-de-l'Île-de-Montréal – Installation Hôpital Maisonneuve-Rosemont
Han Ting Wang (PI), François Marquis (Co-I), Francis Toupin (Co-I), Marc Brosseau (Co-I), Danae Tassy (RC), Lotthida Inthanavong (RA)

Centre hospitalier affilié universitaire régional - CIUSSS de la Mauricie-et-du-Centre-du-Québec
Jean-Nicolas Dubé (PI), Marie-Josée Bériault (Co-I), Marco Chacon (Co-I), David Claveau (Co-I), Jean-François Naud (Co-I), Élise Rodrigue (Co-I), Emmanuel Charbonney (Co-I), Ying Tung Sia (Co-I), Danielle Tapps (RC), Guylaine Toupin (RC)

McGill University Health Centre (Royal Victoria Hospital and Montreal General Hospital)
Jason Shahin (PI) Kosar Khwaja (Co-I), Josie Campisi (RC), Norine Alam (RC)

CIUSSS du Nord-de-l'Île-de-Montréal - Hôpital du Sacré-Coeur de Montréal
Emmanuel Charbonney (PI), Martin Albert (Co-I), Francis Bernard (Co-I), Alexandros Cavayas (Co-I), Karim Serri (Co-I), Virginie Williams (RC), Julia Lainer Palacios (RA), Maxime St-Denis (RA)

Kingston General Hospital
David Maslove (PI), Gordon Boyd (Co-I), John Drover (Co-I), John Muscedere (Co-I), Stephanie Sibley (Co-I), Miranda Hunt (RC), Tracy Boyd (RC), Danielle Muscedere (RA)

The Ottawa Hospital (General Campus and Civic Campus)
Andrew Seely (PI), Shane English (Co-I), Hilary Meggison (Co-I), Guiseppe Pagliorello (Co-I), Rakesh Patel (Co-I), Irene Watpool (RC), Rebecca Porteous (RC), Sydney Miezitis (RA)

Hamilton Health Sciences - Juravinski Hospital
Bram Rochwerg (PI), Tina Millen (RC)

Hamilton Health Sciences - Hamilton General Hospital
Émilie Belley-Côté (PI), Maureen Meade (Co-I), Richard Whitlock (Co-I), Nevena Savija (RC)

St-Joseph's Healthcare
Deborah Cook (PI), Waleed Al-Hazzani (Co-I), Erick Duan (Co-I), Mark Soth (Co-I), Tania Ligori (Co-I), France Clarke (RC), Neala Hoad (RC)

Sunnybrook Health Sciences Centre
Neill KJ Adhikari (PI), Andre Carlos Amaral (Co-I), Brian Cuthbertson (Co-I), Robert Fowler (Co-I), Damon Scales (Co-I), Nicole Marinoff (RC), Navjot Kaur (RC)

University of Alberta Hospital
Oleksa Rewa (PI), Sean Bagshaw (Co-I), Michael Meier (Co-I), Wendi Sligl (Co-I), Nadia Baig (RC), Lorena McCoshen (RC)

Abbreviations:
Co-I – co-investigator
PI – principal investigator
PL – project leader
RA – research assistant
RC – research coordinator
