# Supplementary material for: Lessening Organ dysfunction with VITamin C (LOVIT): protocol for a randomized controlled trial
Source: Trials. 2020 Jan 8;21:42. doi: 10.1186/s13063-019-3834-1 (PMC6950903; doi:10.1186/s13063-019-3834-1)
Supplement: Supplementary file 3 — Additional file 3. Model informed consent form. [file 13063_2019_3834_MOESM3_ESM.pdf]

CENTRE DE RECHERCHE | CHUS

RESEARCH INFORMATION AND CONSENT FORM

**Study Title:**	Lessening Organ Dysfunction with Vitamin C: LOVIT Trial
**Study Number:**	MP-31-2019-2945
**Funding Agency:**	Lotte and John Hecht Memorial Foundation
**Principal Investigator:**	Dr. François Lamontagne, Intensivist
**Co-Investigators:**	Dr. Frédérick D'Aragon, Intensivist<br>Dr. Charles St-Arnaud, Intensivist<br>Dr. Michaël Mayette, Intensivist<br>Pr. Marie-Claude Battista, Scientific Director, Unité de Recherche Clinique et Épidémiologique

FOR INFORMATION

Monday through Friday, from 8 am and 4 pm, you can reach:

Dr. François Lamontagne, Intensivist	Tel: 819-346-1110 ext. 74974
Élaine Carbonneau, Research Nurse	Tel: 819-346-1110 ext. 16208
Marilène Ladouceur, Research Auxiliary Nurse	Tel: 819 346-1110 ext. 14169
Joannie Marchand, Research Assistant	Tel: 819 346-1110 ext. 14171
Marie-Hélène Masse, Research Coordinator	Tel: 819-346-1110 ext. 14173

or dial "0" and ask the operator to call them on pager # 7125.

We are seeking your participation (or that of your family member) in a research study because you (or your family member) have been admitted to an intensive care unit and are treated for a serious infection. However, before you agree to participate, please take the time to read, understand and carefully consider the following information. If you agree to take part in this research study, you will be asked to sign the consent form at the end of this document and we will give you a signed copy for your own records.

This Information and Consent Form explains the goals, procedures, risks, inconveniences and benefits of the study as well as providing the names of the people to reach if needed. This document may contain information or words that you do not understand. Please ask the study investigator or members of the study staff to answer your questions and explain any word or information you do not understand.

NATURE AND GOALS OF THE RESEARCH STUDY

This study aims to determine whether the administration of vitamin C in patients admitted to an intensive care unit (ICU) with a serious infection improves their health status. The current options to treat infections are limited to antibiotics and supportive care for bodily functions (like hydration fluids in the veins, medication to maintain blood pressure, mechanical ventilation and treatment to ensure kidney function). Recent studies suggest that high doses of vitamin C given into veins may be the first therapy helping the body to fight serious infections.

The goal of this study is to determine if the administration of vitamin C decreases the harmful effects of the infection on organs and improves your health status more rapidly than placebo. The specific objectives are to evaluate the effects of vitamin C on your health status and quality of life at 6 months and on blood levels of biomarkers that reflect cellular health. We wish to include approximately 100 participants at the *CIUSSS de l'Estrie - CHUS* to be among the 800 participants needed for this study that will be carried out in 25 hospital centers in Canada. This study will be conducted over 4 years.

Our research team has determined that you are eligible to participate in our study, and your treating physician agrees.

RESEARCH STUDY PROCEDURES

If you agree to participate in this study, you or your family member, will be assigned to either the group to whom we administer vitamin C or to the group to whom we administer a placebo (an inactive substance that has the same appearance that the study product) in the veins. Your assignment to one of these two groups will be determined randomly by a computer that will not retain information about you. The probability of being assigned to either group is 50% (1 in 2 chances or half-and-half). The treating team will not be aware of the group to which you have been assigned, but the doctor in charge of this study can obtain this information rapidly in case of emergency.

As a study participant, you will receive the study product (vitamin C or placebo) 4 times per day during 96 hours or up to your discharge from the intensive care unit whichever comes first. Also, on days 1, 3 and 7 of participation (or when you are discharged from the intensive care unit if it happens before day 7), the nurse will collect 10 ml of urine (2 teaspoons) and 25 ml of blood (5 teaspoons) samples while taking the blood samples required for your regular medical care. We will collect a little more volume than what is needed to compensate for unexpected losses that may arise during laboratory testing. These samples will enable us to measure certain biomarkers in your blood that help assess the function of body's cells and organ systems.

During your hospital stay, we will monitor your progress to see if your organs are functioning well and if you develop other health problems, and to see how long you stay in the ICU and hospital. Your medical chart will be reviewed by the investigator and the research team as long as you remain in the study. Blood test results and procedures present in your medical record will be collected for the study.

After you are discharged from the hospital, you will be contacted by telephone 6 months after the start of your participation in the study. Your contact information will be provided to the Study Coordinating Centre located at the Centre de recherche du Centre hospitalier universitaire de Sherbrooke.

FUTURE ANALYSES

Once the biomarker analyses have been performed as part of this study, it is possible that parts of your samples haven't been used. We wish to use the remainder of your samples to answer additional questions concerning the impact of vitamin C that may arise in future. For example, we may measure a new, as yet undefined, biomarker. Only the remainder of your samples will be used and no other additional sample will be collected.

RISKS ASSOCIATED WITH PARTICIPATION IN THIS RESEARCH STUDY

Vitamin C is generally not toxic. Risks associated to vitamin C are nausea, vomiting, abdominal cramps, tiredness, hot flashes, headaches, insomnia, drowsiness and diarrhea. When vitamin C is administered too rapidly, there is a risk of weakness and dizziness. There is very little probability that these happen since vitamin C will be administered on a 30 to 60 minutes period each time a dose is given. There is a possibility of developing a clot in a deep vein but the simple fact of lying in bed for a long time can cause a clot.

A potential risk is the formation of kidney stones. Vitamin C might increase, decrease or not affect the risk of kidney stones. Whatever the effect on the risk of kidney stones, you might not have symptom or a deterioration of your kidney function can occur, but be counter balanced by a decrease of global mortality. We will nonetheless monitor damage that could harm your kidneys.

Vitamin C may be associated with an exaggerated destruction of red blood cells (hemolysis) in patients with G6PD enzyme deficiency. However, these patients are excluded from participating in the trial. Patients affected by G6PD deficiency are very rare in Canada comparatively to other countries in the world. If hemolysis happens to you during the intervention period, we will stop the study drug and provide standard supportive care.

Vitamin C can give falsely high blood sugar results when it is measured with certain devices used at the bedside. This problem does not occur with equipment used in the hospital laboratory. If a falsely high blood sugar result from a bedside device occurred, your doctor may decide to use insulin to lower your blood sugar, which may cause low blood sugar (hypoglycaemia). In this study, various measures are taken to minimize the risk of false blood sugar levels, including the use of bedside devices that are more reliable than others, and more frequent blood tests for blood sugar to be measured in the hospital laboratory. Accordingly, this study may be associated with more frequent blood tests.

RISKS ASSOCIATED TO THE PROCEDURES

The risks associated to blood samples are the following: mild pain, dizziness, fainting, bruising, bleeding and in rare cases blood clots and infection. However, you or your family member will have already a catheter in place that allows for blood samples to be drawn without a puncture (and then not exposed to the risks mentioned above).

RISKS ASSOCIATED WITH PREGNANCY

Taking part in this research study may include known or unknown risks to pregnant women, embryos, foetuses, or breastfed infants. Therefore, pregnant or breastfeeding women cannot participate in this research study.

BENEFITS ASSOCIATED WITH YOUR PARTICIPATION IN THE RESEARCH STUDY

You might have a personal benefit to participate in this research study, but we can't guarantee. However, the findings from this study may help increase our knowledge of infections and vitamin C administration, which may benefit future patients.

VOLUNTARY PARTICIPATION AND THE RIGHT TO WITHDRAW

Your participation in this research study is voluntary. Therefore, you may refuse to participate. You can also withdraw from the study at any time, without providing a reason, by informing the study investigator or one of his assistants.

Your decision not to participate in the study or to withdraw from it will have no impact on the quality of care and services you are entitled to or on your relationship with the investigator and other stakeholders.

The study investigator, the funding agency or the research ethics board may end your participation in the study. This may happen if new findings or information indicates that participation is no longer in your interest; if the study investigator believes it is in your best interest; or if there are administrative reasons to terminate the study.

If you withdraw or are withdrawn from the study, the information and material already collected during the course of the study will be stored, analyzed or used to protect the scientific integrity of the study.

Any new study findings that could influence your decision to stay in the research study will be shared with you as soon as possible.

CONFIDENTIALITY

While you take part in this research project, the study investigator and study staff will collect and record information about you in a study file. They will only collect information required to meet the scientific goals of the study.

This study file may include information from your medical chart concerning your past and present health status, your lifestyle as well as the test results, exams and procedures you will undergo during the study.

Study samples will be sent to the Centre de recherche du Centre hospitalier universitaire de Sherbrooke, under the responsibility of Dr. François Lamontagne, and kept until the end of the study and the analyses for this study exclusively and then destroyed.

All the information collected during the study will remain strictly confidential to the extent provided by law. You will be identified by an alphanumeric code. The key to the code linking your name to your study file will be kept in a safe place by the doctor in charge of this research study.

Your full name and your telephone number will be transmitted to a qualified person of the Study Coordinating Centre to allow this person to contact you in 6 months by telephone. We will also ask you (or your family member) the name and phone number of alternative contacts to ensure we facilitate the telephone follow-up in 6 months. This personal information will allow a direct identification. This information will be kept in security and confidentiality will be preserved by the qualified person of the Study Coordinating Centre and destroyed at the end of the follow-up.

To ensure your safety, a mention of your participation in this research study will be included in your medical file. Therefore, any person or company to whom you will give access to your medical file will have access to this information.

The doctor responsible of this study will send to the principal investigator of the study, the coded data concerning your participation.

The research data will be stored by the study investigator for at least 25 years.

Study results may be published in medical journals or discussed at scientific meetings, but it will be impossible to identify you.

For monitoring and control purposes, your study file and medical records may be examined by a representative of the Research Ethics Board of the institution or by a person mandated by a regulatory authority, for example, Health Canada. Data from your study file will be sent to the Data Management Centre located within the Department of Health Research Methods, Evidence, and Impact at McMaster University. All these individuals and organizations adhere to confidentiality policies.

You have the right to consult your study file at any time to verify the information gathered and to have it corrected, if necessary, for as long as this information is available to the study investigator or the institution. However, some of this information may be made available to you only once the study has ended, to protect the scientific integrity of the study.

COMPENSATION

You will not receive any compensation for expenses and inconveniences incurred due to your participation in this research study.

SHOULD YOU SUFFER ANY HARM

Should you suffer any harm due to your participation in this research study, you will be provided with all the necessary care and services, at no cost to you.

By agreeing to take part in this study, you are not waiving any of your legal rights nor discharging the study investigators, the sponsor or the institution where this research study is being conducted of their civil liability and professional responsibilities.

CONTACT PERSONS

If you have any questions or problems related to the research study or if you wish to withdraw from it, you can contact the research team. Please refer to the box on page 1.

If you have any questions regarding your rights as a participant in this study or if you have any complaints, you may contact the CIUSSS de l'Estrie-CHUS' Office of Complaints and Quality of Services at plaintes.ciussse-chus@ssss.gouv.qc.ca or at the following number: 1-866-917-7903.

MONITORING OF ETHICAL ASPECTS OF THE STUDY

The Research Ethics Board of the *CIUSSS de l'Estrie - CHUS* approved this study and is in charge of its monitoring for the participating institutions of the Québec Health and Social Services Network.

If you wish to contact a member of that board, you can reach the Research Ethics Support Services of the *CIUSSS de l'Estrie - CHUS* at ethique.recherche.ciussse-chus@ssss.gouv.qc.ca or at the following number: 819-346-1110, ext. 12856.

CONSENT

I declare that I have read this Information and Consent Form. I declare that the research study has been explained to me, that my questions were answered to my satisfaction and that I was given sufficient time for consideration and to make a decision. Upon reflection, I agree to participate in this research study under the conditions stated therein.

I accept that the research team will have access to my medical record.

I agree that the remainder of the samples may be used for additional analyses that may arise during the study (future analyses). ☐ **YES** ☐ **NO**

Name of participant *(please print)*	Signature of participant	Date

I have explained the research study and this Information and Consent Form and I have answered all of his/her questions.

Name of person obtaining consent *(please print)*	Signature of person obtaining consent	Date

CONSENT FROM REPRESENTATIVE (SUDDEN INCAPACITY)

Because Mr./Mrs.______________________ has suddenly become incapable of giving consent for the hereinafter mentioned reason, the Civil Code of Québec allows you to give consent for him/her as his/her _______________________ (indicate your relationship with the participant).

As soon as Mr./Mrs. ______________________has sufficiently recovered, he/she will be asked to sign his/her own consent form to indicate whether he/she wants to continue taking part in this study. I accept that the research team will have access to his/her medical record

REASON WHY THE PARTICIPANT CAN NOT CONSENT:

____

By signing this page, I confirm that I have read the information in this Consent Form. I acknowledge that the study has been explained to me, that all of my questions have been answered and that I was given enough time to make a decision. I voluntarily give my consent so that ___________________ can participate in this study.

I accept that the research team will have access to his/her medical record.

I also agree that the remainder of the samples may be used for additional analyses that may arise during the study (future analyses). ☐ **YES** ☐ **NO**

Name of representative *(please print)*	Signature of representative	Date

I have explained the research study and this Consent Form to the participant's legal representative. I have answered all of his/her questions.

Name of person obtaining consent *(please print)*	Signature of person obtaining consent	Date

CONSENT FROM THE LEGAL REPRESENTATIVE OR CAREGIVER SUPPORTING THE PARTICIPATION OF THE PERMANENTLY INCAPABLE PARTICIPANT (PERMANENT INCAPACITY)

I declare that I have read this Information and Consent Form. I declare that the research study has been explained to me, that my questions were answered to my satisfaction and that I was given sufficient time for consideration and to make a decision.

I agree that __________________ can participate in this research study under the conditions stated therein. I will receive a signed and dated copy of this Information and Consent Form.

I accept that the research team will have access to his/her medical record.

I also agree that the remainder of the samples may be used for additional analyses that may arise during the study (future analyses). ☐ **YES** ☐ **NO**

If the incapacitated participant is represented:

____

Name and signature of the legal representative (tutor, curator or mandatary) — Date

If the incapacitated participant is not represented by a legal representative:

____

Name and signature of the spouse, failing which, name of next-of-kin or name of a significant person — Date

I have explained the research study and this Consent Form to the participant's legal representative. I have answered all his/her questions.

____

Name of person obtaining consent *(please print)*	Signature of person obtaining consent	Date

TELEPHONE CONSENT
(For the participant who is suddenly or permanently incapacitated)

Because ______________________ is incapable of giving consent for the hereinafter mentioned reason,

I have explained the research study and this Consent Form to the participant's legal representative. I have answered all his/her questions. I accept that the research team will have access to his/her medical record.

The representative, Mr./Mrs. ____
Name of the legal representative (tutor, curator or mandatary)
Name of the spouse or next-of-kin or
Name of the significant person

has given consent by telephone on ___________________ at_______________
Date Hour

The representative accepts that the research team will have access to his/her medical record.

The representative also agrees that the remainder of the samples may be used for additional analyses that may arise during the study (future analyses**).** ☐ **YES** ☐ **NO**

I have explained to the representative the research project and this information and consent form and I have answered to his/her questions.

Name of person obtaining consent *(please print)*	Signature of person obtaining consent	Date
