# Supplementary material for: Lessening Organ dysfunction with VITamin C (LOVIT): protocol for a randomized controlled trial
Source: Trials. 2020 Jan 8;21:42. doi: 10.1186/s13063-019-3834-1 (PMC6950903; doi:10.1186/s13063-019-3834-1)
Supplement: Supplementary file 4 — Additional file 4. Authorship and substudy guidelines. [file 13063_2019_3834_MOESM4_ESM.pdf]

LOVIT AUTHORSHIP AND SUB-STUDY GUIDELINES

Version: 1.0

Date: 10-Oct-2018

The LOVIT Authorship and Sub-Study Guidelines are the confidential intellectual property of the LOVIT Principal Investigators and LOVIT Steering Committee and cannot be used in any form without the expressed permission of the Principal Investigators.

DOCUMENT REVISION HISTORY

Date	Version Number	Section(s) Affected	Summary of Change(s)	Author(s)
		Entire Document	Original Version	

1.0. INTRODUCTION

This document describes the policies for the protocol and primary manuscripts, sub-studies, and secondary analyses and manuscripts. The LOVIT Principal Investigators encourage co-investigators to develop ideas for sub-studies to be conducted alongside the LOVIT trial and to submit proposals for secondary analyses and corresponding manuscripts using the LOVIT data. Sub-studies are defined as studies that build on the LOVIT trial infrastructure and require additional data collection. Secondary analyses and manuscripts use data that are collected as part of the LOVIT trial to address a research question that is not included in the LOVIT protocol.

2.0 PROTOCOL MANUSCRIPT AND PRIMARY MANUSCRIPT

We will use named authorship (as opposed to group authorship) for the LOVIT protocol publication and primary LOVIT results publications (**Figure 1**). Under the direction of the Principal Investigators, the named co-authors will be responsible for the preparation of the manuscripts. The Executive Committee will collaboratively determine named co-authors based on the tool in **Table 1**. The Principal Investigators envision many named co-authors for both the protocol manuscript and the primary manuscript. To be a named co-author, individuals must also meet the ICMJE authorship requirements (**Table 2**).

The order of authorship will be based on the level of contribution to the LOVIT trial, as determined by the Executive Committee, with support from the Steering Committee membership as needed. The tool in **Table 1** may also be used to determine authorship order.

Table 1: Tool for Determining Named Co-Authors and Authorship Order

Item	Specify	Level of Engagement or Contribution	Comments
Leadership role(s) in trial	- Active member of the Steering Committee<br>- Country lead<br>- Other committee member role (specify)<br>- Coordinating Centre role (specify)<br>- Other leadership role (specify)	- Very high<br>- High<br>- Moderate<br>- Low<br>- Very low	
Clinical site leadership	- Patient enrolment<br>- Data quality<br>- Patient follow-up	- Very high<br>- High<br>- Moderate<br>- Low<br>- Very low	
Expertise provided	- Design<br>- Methodology<br>- Clinical<br>- Statistical<br>- Other (specify)	- Very high<br>- High<br>- Moderate<br>- Low<br>- Very low	

Item	Specify	Level of Engagement or Contribution	Comments
Drafted sections of the manuscript		- Very high<br>- High<br>- Moderate<br>- Low<br>- Very low	
Provided input on the interpretation of the data		- Very high<br>- High<br>- Moderate<br>- Low<br>- Very low	
Critically reviewed and edited the manuscript		- Very high<br>- High<br>- Moderate<br>- Low<br>- Very low	

Table 2: ICMJE Authorship Requirements (available at http://icmje.org/)

1. Substantial contributions to the conception or design of the work; or the acquisition, analysis, or interpretation of data for the work; AND
2. Drafting the work or revising it critically for important intellectual content; AND
3. Final approval of the version to be published; AND
4. Agreement to be accountable for all aspects of the work in ensuring that questions related to the accuracy or integrity of any part of the work are appropriately investigated and resolved.

In addition to being accountable for the parts of the work he or she has done, an author should be able to identify which co-authors are responsible for specific other parts of the work. In addition, authors should have confidence in the integrity of the contributions of their co-authors.

The Principal Investigators will take the lead on preparing the first drafts of both the protocol manuscript and the primary manuscript. They will ask named co-authors to draft sections of the manuscripts, provide clinical or methodological expertise, and critically review the manuscripts in their entirety.

For the protocol manuscript and the primary manuscripts, all members of the LOVIT team will be listed in the contributor appendix (see Section 5.0). Journal rules on listing contributors will be followed in an effort to ensure that all contributors are searchable through PubMed.

Manuscript Title

Author A, Author B, Author C on behalf of the LOVIT Investigators
and the Canadian Critical Care Trials Group*

Appendix

Executive Committee

Steering Committee

Data and Safety Monitoring Board

Coordinating Centre

Participating Clinical Sites

Figure 1: Authorship for the Protocol Manuscript and the Primary Manuscript

3.0 SUB-STUDIES

LOVIT co-investigators may submit ideas for sub-studies that build on the existing infrastructure and data collected within the LOVIT trial. LOVIT co-investigators should submit proposals for sub-studies to the Principal Investigator(s) or Project Manager(s) at the Coordinating Centre. All ideas for sub-studies will be archived.

The proposals should include:

- Background and rationale
- Research question(s)
- Methods
- Statistical analysis plan (if developed)
- Timeline
- Implementation plan
- Impact on the LOVIT trial
- Budget and sources of funding

The Principal Investigators and Project Managers, with input from the Steering Committee and larger LOVIT team as needed, will determine which sub-studies will be accepted. To do so, the Principal Investigators will complete the tool in **Table 3** to help guide their decision. The Principal Investigators may also request members of the Steering Committee review the proposals and complete the below tool, as needed. As a general rule, sub-studies should not compromise the likelihood of successfully completing the main trial within set timelines.

If multiple co-investigators submit the same (or similar) ideas for sub-studies, the Principal Investigator(s), with support from the Steering Committee as needed, will select

a co-investigator to take the lead on the sub-study. This selection will be based on knowledge of the topic proposed, availability of the required resources (e.g. co-investigator time) to devote to the sub-study, and contributions to the LOVIT trial (e.g. Steering Committee member, volume of patients enrolled at clinical site, etc.). The checklist tool in **Table 3** may be used to guide this decision. Co-investigators who submitted similar ideas may be invited to contribute as co-investigators on the sub-study at the discretion of the Principal Investigators and the lead co-investigator. If a lead co-investigator is unable to devote sufficient time and resources to a sub-study, the Principal Investigators, with guidance from the study team, may recommend that another co-investigator lead the sub-study.

Table 3: Tool for Determining Which Sub-Studies to Approve

Item	Strongly Disagree	Disagree	Neutral	Agree	Strongly Agree	Comments
Sub-study addresses an important, clinically relevant, and timely research question						
Sub-study is feasible and can be completed						
Sub-study will have a positive impact on the LOVIT trial						
There are adequate funding and resources to complete the sub-study						
Co-investigator is on the Steering Committee (Yes/No)						
Co-investigator is from a high enrolling clinical site						
Data is of high quality at the co-investigator's clinical site						
Co-investigator actively participates in LOVIT trial meetings						

The Principal Investigators, and members of the Executive Committee (and Steering Committee as needed), will meet and review the sub-study proposals and their responses each of the above questions. They will collaboratively determine which proposals to accept via consensus. Once a decision has been made, the Principal Investigators will communicate the decision to the co-investigator who submitted the sub-study proposals. If a proposal is not accepted, a Principal Investigator may telephone the co-investigator to explain the rationale for not moving forward with the proposed sub-study.

The Principal Investigators, the Project Managers at the Coordinating Centre, and members of the LOVIT team (as needed), will work with the co-investigators on accepted sub-study proposals to develop the sub-study protocol and incorporate it into the LOVIT trial infrastructure. They will also provide oversight and support, as needed, to the conduct of the sub-study.

For sub-study LOVIT publications, we will typically credit authorship those who led and participated in the sub-study, with acknowledgment to the rest of the group, i.e., on behalf of the LOVIT Investigators (**Figure 2**). All named authors will be expected to adhere to ICMJE authorship criteria; it is anticipated that the LOVIT Principal Investigators will qualify for authorship. The tool found in **Table 1** may be used to for determining guide authorship and all authors must meet the criteria in **Table 2**. All other contributors will be listed in the contributor appendix and every effort will be made to ensure that all contributors are searchable through PubMed.

Sub-Study Manuscript Title

Author A, Author B, and Author C on behalf of the LOVIT Investigators

Appendix

Executive Committee

Steering Committee

Data and Safety Monitoring Board

Coordinating Centre

Participating Clinical Sites

Figure 2: Authorship for Sub-Study Manuscripts

4.0 SECONDARY ANALYSES AND MANUSCRIPTS

We encourage LOVIT co-investigators to conduct/oversee secondary analyses and to write the corresponding secondary manuscripts using the data that is collected within the LOVIT trial. LOVIT co-investigators should submit proposals for secondary analyses

and corresponding manuscripts to the Principal Investigators or Project Managers at the Coordinating Centre.

The proposals should include:
- Background and rationale
- Research question(s)
- Proposed methods
- Statistical analysis plan (if developed)
- Timeline
- Requirement of Coordinating Centre resources (e.g. statistical analysis support)
- Funding / resources

The Principal Investigators and Project Managers, with input from the Executive Committee and Steering Committee as needed, will review the proposal and determine if it is acceptable. They may use the checklist tool in **Table 4** to help make this determination. They may request additional details and clarification from the co-investigator if the any aspect of the proposal is unclear. They may also request revisions to the proposal. The Principal Investigators may also deny a request for a secondary analysis, and if so, they will provide a rationale to the co-investigator who submitted it.

If multiple co-investigators submit the same (or similar) ideas for secondary analyses and corresponding manuscripts, the Principal Investigator(s), with support from the Executive and Steering Committees as needed, will select a co-investigator to take the lead on the analysis and manuscript preparation. This selection will be based on: knowledge of the topic proposed, availability of the required resources (e.g. co-investigator time) to complete the secondary analysis and corresponding manuscript in a timely fashion, and contributions to the LOVIT trial (e.g. Steering Committee member, volume of patients enrolled at clinical site, etc.). The checklist tool in **Table 4** may be used to guide this decision. Co-investigators who submitted similar ideas may be invited to contribute as co-authors at the discretion of the Principal Investigators and the lead co-investigator. If a lead co-investigator is unable to complete the planned analyses and secondary paper in a timely fashion, the Principal Investigators, with guidance from the study team, may recommend that another co-investigator take the lead.

Table 4: Tool for Determining Which Secondary Analyses and Manuscripts to Approve

Item	Strongly Disagree	Disagree	Neutral	Agree	Strongly Agree	Comments
Analyses addresses an important, clinically relevant, and timely research question						
Analysis is feasible and can be completed						

Item	Strongly Disagree	Disagree	Neutral	Agree	Strongly Agree	Comments
Analysis will have a positive impact on the LOVIT trial						
Analysis is different enough from other proposed secondary analyses						
There are adequate funding and resources to complete the analysis						
Co-investigator is on the Steering Committee (Yes/No)						
Co-investigator is from a high enrolling clinical site						
Data is of high quality at the co-investigator's clinical site						
Co-investigator actively participates in LOVIT trial meetings						

The LOVIT study statistician will work with the lead co-investigator and members of the study team to develop the statistical analysis plan. In most situations, the statistical analyses will be conducted by the LOVIT statistician. If the analysis is not conducted by the LOVIT statistician, the LOVIT Principal Investigators and statistician will review the statistical analysis plan. They will also determine which data fields to send to the co-investigator for analysis.

The Principal Investigators will also review draft manuscripts and they will approve the final manuscript prior to journal submission to ensure that the manuscript is of acceptable quality.

The Principal Investigators may also ask members of the LOVIT co-investigators to lead LOVIT secondary analyses and manuscripts. This selection will be based on the knowledge and interest of a topic and contributions to LOVIT trial. The applicable processes, as described above, will be followed.

Coordinating Centre personnel may assist with the secondary analysis and manuscript preparation, as needed. The LOVIT Coordinating Centre Project Managers will maintain a listing of all secondary analyses and manuscripts including the lead co-investigator, co-authors, and the status of the analysis and manuscript. Additionally, the Principal Investigators, with input from the Steering Committee and Project Managers, may prioritize the order in which the secondary analyses are conducted. The Principal Investigators are responsible for ensuring the quality of the secondary manuscripts and that the messaging of the secondary manuscripts is not conflicting.

For secondary LOVIT publications, we will credit authorship to the individual co-investigators who write the secondary manuscripts, with acknowledgment to the rest of the group, i.e., on behalf of the LOVIT Investigators (**Figure 3**). The lead investigator for each secondary paper, with support from the Principal Investigators, will determine named co-authors. Named co-authors will include the Principal Investigators and members of the LOVIT team who actively contribute to the publication per ICMJE authorship criteria. The tool in **Table 1** may be used to for determining guide authorship and all authors must meet the criteria in **Table 2**. All other contributors will be listed in the contributor appendix and every effort will be made to ensure that all contributors are searchable through PubMed.

Secondary Manuscript Title

Author A, Author B, and Author C on behalf of the LOVIT Investigators

Appendix

Executive Committee

Steering Committee

Data and Safety Monitoring Board

Coordinating Centre

Participating Clinical Sites

Figure 3: Authorship for Secondary Manuscripts

5.0. DEVELOPMENT OF THE CONTRIBUTOR APPENDIX

The contributor appendix for the LOVIT manuscripts will acknowledge the:

- Executive Committee Members
- Steering Committee Members
- Data and Safety Monitoring Board Members
- Coordinating Centre Personnel
- Participating Clinical Sites Personnel

Within each participating clinical site, the following individuals will be listed:

- Site Principal Investigator
- Site Sub-Investigators
- Study Coordinators

The order of clinical sites listed in the contributor appendix will be based upon geographic region. Within each geographical region, the order of the clinical sites will be based on participant enrollment, in the order of highest enrollment to the lowest enrollment.

We will credit investigators for their contribution while they were at a LOVIT site if they relocate to a hospital that is not participating in LOVIT. If an investigator relocates to another LOVIT site or starts a new LOVIT site, we will acknowledge them for their contributions at both sites.

Finally, we will include clinical sites who are initiated and did not enroll participants in the contributor appendix. These clinical sites will be listed under a sub-heading of initiated and did not enroll.

The Coordinating Centre Project Manager(s) will be responsible preparing and maintaining the contributor appendix. To do so, they will contact each site to confirm the individuals who should be listed. The Project Managers will circulate the contributor list to each individual clinical site to ensure its accuracy. It is the site’s responsibility to ensure that all participating members at their site are listed and that spelling and credentials are correct.
